# Subjective and Objective Mental and Physical Functions Affect Subjective Cognitive Decline in Community-Dwelling Elderly Japanese People

**DOI:** 10.3390/healthcare8030347

**Published:** 2020-09-18

**Authors:** Akio Goda, Shin Murata, Hideki Nakano, Kayoko Shiraiwa, Teppei Abiko, Koji Nonaka, Hiroaki Iwase, Kunihiko Anami, Jun Horie

**Affiliations:** 1 Department of Physical Therapy, Faculty of Health Sciences, Kyoto Tachibana University, Kyoto 607-8175, Japan; murata-s@tachibana-u.ac.jp (S.M.); nakano-h@tachibana-u.ac.jp (H.N.); shiraiwa@tachibana-u.ac.jp (K.S.); abiko@tachibana-u.ac.jp (T.A.); horie-j@tachibana-u.ac.jp (J.H.); 2 Department of Rehabilitation, Faculty of Health Sciences, Naragakuen University, Nara 631-8524, Japan; nonaka@naragakuen-u.jp (K.N.); anami@naragakuen-u.jp (K.A.); 3 Department of Physical Therapy, Faculty of Rehabilitation, Kobe International University, Kobe 658-0032, Japan; iwase@kobe-kiu.ac.jp

**Keywords:** community-dwelling older adults, mental function, physical function, preclinical Alzheimer’s disease, subjective cognitive decline

## Abstract

Subjective cognitive decline (SCD) is complex and not well understood, especially among Japanese people. In the present study, we aimed to elucidate the relationships of subjective and objective mental and physical function with SCD among older community-dwelling Japanese adults. SCD was evaluated using the Kihon Checklist: Cognitive Function. Other parameters were evaluated using the Mini-Mental State Examination (MMSE) and the five-item version of the Geriatric Depression Scale (GDS-5), for an objective mental function other than SCD. A timed up-and-go test (TUG) and knee extension strength were used to test objective physical function, and the Mental Component Summary (MCS) and Physical Component Summary (PCS) in the Health-Related Quality of Life survey eight-item short form (SF-8) were used for subjective mental and physical functions. The results of the MMSE, GDS-5, TUG, knee extension strength, and MCS were significantly worse in the SCD group. In addition, logistic regression analysis showed that GDS-5 and MCS were associated with SCD onset. Depressive symptoms and decreased subjective mental function contribute to SCD among community-dwelling Japanese adults. These findings will be useful for planning dementia prevention and intervention programs for older Japanese adults.

## 1. Introduction

The Japanese population is aging rapidly, and the incidence of dementia is thus increasing [1]. Dementia is associated with a high risk of disability and, consequently, death in older adults [2]. Therefore, there is an urgent need to develop treatment strategies for dementia and Alzheimer’s disease (AD). However, almost all recent clinical trials of drugs aimed at treating AD have been unsuccessful [3]. Hence, until effective therapeutic agents are developed, it is important to elucidate the risk factors for dementia onset and adopt interventions, where possible, to change lifestyle and individual patient’s characteristics [4]. In recent years, to prevent and delay the progression of pathological cognitive decline in older adults, it has been recommended that intervention be initiated at an early stage of AD, such as at the preclinical phase [5].

In this context, subjective cognitive decline (SCD) has gained increasing attention as a typical symptom of preclinical AD [6]. Individuals with SCD have reported a decline in acquired cognitive function despite normal performance in objective cognitive function tests and absence of any difficulty with activities of daily living [7]. SCD is predictive of objective cognitive decline [8,9] and the onset of mild cognitive impairment [10] and dementia [10,11] in older people who do not have cognitive impairment. Thus, it is considered an indicator of abnormal cognitive decline. In addition to objective mental function—which includes cognitive function [12,13], depression [12,13,14,15,16,17,18,19,20,21], and anxiety [12,13,22,23]—there are other factors associated with SCD which include objective physical function [24,25], subjective mental function [26,27], and subjective physical function [28,29], as well as age [15] and educational history [15]. SCD is thus complex and is not fully understood [28]. In addition, the bulk of published literature on SCD is biased toward Europe and the USA, and it has been reported that risk factors for cognitive decline have different intensities in Japanese people compared to Europeans and Americans [30]. It is therefore, unclear whether research results of factors that affect SCD in Europeans and Americans will be directly applicable to the Japanese population. Admittedly, almost no research has been performed on factors that affect SCD in Japanese people, and this issue has not yet been resolved. In summary, the factors that affect SCD in Japanese people are unclear and may differ from those shown in previous studies involving Europeans and Americans, in which case appropriate interventions for preventing dementia may also differ between these groups.

In the present study, in line with previous research, we hypothesized that SCD in Japanese people is associated with subjective and objective mental and physical function. To test this hypothesis, we investigated the relationships between SCD and subjective and objective mental and physical function in community-dwelling older adults in Japan.

## 2. Materials and Methods

This study included a cross-sectional survey of community-dwelling older adults, and the measurements were performed in September 2014 and 2015. Participants were recruited through fliers distributed in 2014 and 2015, from June to August, in Yasu City, Shiga Prefecture. The fliers mentioned that there would be no compensation for participation. The inclusion criteria we as follows:

(i) Aged over 60 years;
(ii) No warning signs of marked cognitive impairment, with a score of less than 24 in the Mini-Mental State Examination (MMSE);
(iii) No difficulties in walking unaided.

The exclusion criteria were as follows:

(i) Inability to understand instructions in the physical and cognitive tests;
(ii) Previous history of mental illness;
(iii) Cannot complete all measurements.

**Figure 1:**
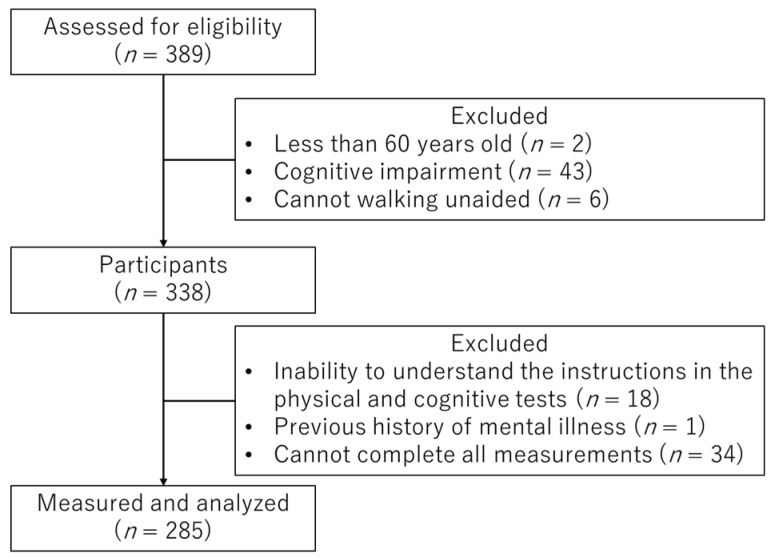
Flowchart of the selection process of the study participants in the present study.

The final analysis used data from 285 participants (Figure 1).

We performed the study in accordance with the Declaration of Helsinki, and written informed consent was obtained from each individual before participation. The study was approved by the Kyoto Tachibana University’s Ethics Committee (approval no.: 14-5).

Several other parameters were evaluated in addition to SCD. Objective mental function was evaluated using MMSE and the five-item version of the Geriatric Depression Scale (GDS-5). Objective physical function was evaluated based on the timed up-and-go test (TUG) time and knee extension strength. Subjective mental function was evaluated using the Mental Component Summary (MCS) in the eight-item short form (SF-8) of the Health-Related Quality of Life survey. Subjective physical function was evaluated using the Physical Component Summary (PCS) of SF-8.

In the interview about SCD, subjects were asked three questions from the Cognitive Function section of the Kihon Checklist (KCL-CF), which is a self-declaration questionnaire on frailty. Its validity [24,31] and reliability [32] have been established. The questions are as follows [32]: “Do your family or your friends point out your memory loss?”; “Do you make a call by looking up phone numbers?”; “Do you find yourself not knowing today’s date?” In previous studies, KCL-CF was used as an index of subjective memory complaints [33], subjective cognitive complaints [34], and self-reported-cognitive decline [35]. In the present study, however, KCL-CF was considered the index of SCD. A score of 1 was given for each answer to indicate a negative state. The total score was calculated, and any subject with a score of 1 or more (KCL-CF ≥ 1) was considered to have SCD.

Global cognitive function was evaluated using MMSE [36]. MMSE is a short test with wide, international use for efficient evaluation of cognitive function and covers 11 areas, including writing letters and copying shapes. This screening tool is valid [37] and reliable [38]. Performance was evaluated based on the total MMSE score covering all areas.

Depressive symptoms were evaluated using GDS-5, a concise version of GDS [39]. GDS is a self-reported screening questionnaire that was prepared with consideration given to the characteristics of depressive symptoms in older adults. This tool has been reported to be valid [39] and reliable [40]. The GDS-5 tool has five questions with a yes or no format. A score of 1 is given for each answer indicating a depressive state, and evaluation is based on the total score.

TUG for objective physical function was performed using the method reported by Podsiadlo et al. [41]. The test started with the subjects seated in a chair at a height of 42 cm with no elbow rests. The subjects were instructed that, when given a sign to start, they should stand up out of the chair, walk to and back from a point 3 m away as quickly as possible, and then sit back in the chair. This test is valid and reliable [41]. The time needed to complete this procedure was recorded and analyzed.

Knee extension strength was measured using the method detailed by Bohannon [42]. Subjects started in the seated position, with their waist and knees bent at angles of 90°. The sensor pad of a hand-held dynamometer (μTasF-1; Anima Corporation, Tokyo, Japan) was pressed against the shin close to the ankle, and the subjects were instructed to make the strongest possible isometric contraction of the quadriceps femoris muscle in one leg. This assessment tool is valid [43] and reliable [44]. A total of four measurements, two for each leg, were made, and the maximum value measured was used in the analysis.

MCS and PCS were measured using SF-8, a self-reporting questionnaire on health-related quality of life developed by Fukuhara et al. [45] SF-8 is a concise version of SF-36 [46], and its validity and reliability have been established [45,47]. SF-8 covers four areas that contribute to MCS—namely vitality, social functioning, role-emotional, and mental health—and four areas that contribute to PCS—namely physical functioning, role-physical, body pain, and general health. MCS and PCS were both calculated from the SF-8 measurements using the linear T-score transformation (mean: 50; standard deviation: 10). They were also calculatedfrom the scoring algorithm based on the 2007 national standard norms added by Fukuhara et al. for further analysis as indices of subjective mental function [48] and subjective physical function [49], respectively. MCS is affected by cognitive and emotional factors, but the effect of the emotional aspect is particularly large [29]. Therefore, MCS is considered an index that strongly reflects the emotional element among mental functions.

For statistical analysis, the normality of data was confirmed for each subject group and evaluation item using the Shapiro–Wilk test. Next, the *t*-test was performed with independent samples for which normality could be confirmed. The Mann–Whitney U-test was then performed with independent samples for which normality could not be confirmed, and the χ^2^ test was conducted to compare the SCD and non-SCD groups. Finally, using a logistic regression model, the degree to which the presence or absence of SCD could be predicted from the subjective and objective mental and physical function was investigated. SPSS Statistics software (version 24; IBM, Armonk, NY, USA) was used for all analyses, with the significance level set at 5%.

## 3. Results

The KCL-CF scores and frequency data for SCD items are shown in Table 1. Regarding the presence or absence of SCD, at least 50% of all subjects replied that they had no symptoms. Positive responses were provided by 35.4% of subjects for at least one SCD item, the proportions being 27.4% for one item, 7.7% for two items, and 0.4% for three items. The question with the highest frequency of affirmative answers was, “Do you find yourself not knowing today’s date?” at 26.7%, and the one with the lowest frequency was “Do you make a call by looking up phone numbers?” at 2.1%.

Table 2 shows demographic variables and descriptive information about the subjective and objective mental and physical functions. An inter-group comparison was made based on the presence or absence of SCD. Significant inter-group differences were found in objective mental function (MMSE and GDS-5), objective physical function (TUG, and knee extension strength), and subjective mental function (MCS). On the other hand, no inter-group differences were found in age, sex, educational history, or subjective physical function (PCS).

The results of logistic regression analysis performed with the presence or absence of SCD as the dependent variable are shown in Table 3. Objective mental function [GDS-5: OR = 1.61; 95% confidence interval (CI) = 1.18 to 2.20] and subjective mental function (MCS: OR = 0.92; 95% CI = 0.87 to 0.98) were found to be significantly associated with the presence or absence of SCD.

## 4. Discussion

The hypothesis proposed in this study was that SCD is associated with subjective and objective mental and physical functions among Japanese people. Subjects with SCD showed significantly worse results for objective mental function (MMSE and GDS-5), objective physical function (TUG, and knee extension strength), and subjective mental function (MCS) than subjects without SCD. In addition, the results of logistic regression analysis indicate that objective mental function (depressive symptoms; GDS-5) and subjective mental function (MCS) were independent factors that influenced the presence or absence of SCD.

The prevalence of SCD (KCL-CF ≥ 1) among subjects in this study was 35.4%. Previously, the prevalence of SCD among Japanese, community-dwelling older adults was reported as 32.5% [34], 34.5% [50], 34.9% [35], and 37.6% [51]. Regarding the response frequencies for the lower order questions, the prevalence of affirmative answers to, “Do your family or your friends point out your memory loss?” and, “Do you find yourself not knowing today’s date?” was high, whereas that for, “Do you make a call by looking up phone numbers?” was low. This tendency was similar to that reported in previous studies among Japanese, community-dwelling older adults [35,50]. Both the SCD prevalence and lower order response frequencies were thus broadly consistent with those reported in previous studies.

The result of a comparison between the groups with and without SCD was that there were significant inter-group differences in objective mental function (MMSE and GDS-5), objective physical function (TUG and knee extension strength), and subjective mental function (MCS). Significant differences between the SCD and non-SCD groups in cognitive function [12,13], depressive symptoms [19,21], and physical function such as mobility and muscular strength [24,25], have been reported in previous studies. In addition, subjective mental function (MCS) is associated with unidentified complaints related to cognitive function in older adults [27] and a decrease in MCS is characteristic of SCD [26]. Therefore, the findings in this study are consistent with those in previous studies. However, no significant inter-group differences were found in subjective physical function (PCS). Previous clinical studies have reported lower PCS in subjects with SCD than in healthy subjects [29]. This inconsistency between our results and those of published literature may be due to differences in levels of negative emotions. Patients with high levels of negative emotions have been reported to pay a lot of attention to their physical symptoms, hence increased dissatisfaction about physical function [52]. The mean MCS in a previous study was 48.44 points [29] and was somewhat lower than that in this study (52.2 points). It is suggested that this difference is because the subjects in the previous study [29] had high levels of negative emotions that resulted in obsession with their own physical symptoms, and thus a marked tendency to under-evaluate their physical function, resulting in low PCS.

Logistic regression analysis adjusted for age, sex, and educational history, with the presence or absence of SCD as the dependent variable, revealed that SCD clearly had an association with the objective mental function (GDS-5) and subjective mental function (MCS). Effects of depressive symptoms on SCD have been reported in numerous previous studies [18,20,22]. As explained above, patients with high levels of negative emotions, as is the case with depression, are likely to concentrate a lot on their physical symptoms, hence the reason for increased dissatisfaction [16,52]. Subjective mental function (MCS) was calculated from lower order scores in SF-8, covering subjective symptoms such as depression, anxiety, and fatigue, and their effects on activities of daily living and social activity. There are reports on the association of SCD with depressive symptoms [13,14,17,22], anxiety [12,13,22,23], and fatigue [53], which is consistent with our finding that MCS is associated with SCD. In addition, based on the results of logistic regression analysis, subjective and objective physical function did not affect the presence or absence of SCD. A previous multivariate analysis showed no relationships between objective physical function, such as mobility and muscular strength, and SCD [24], which is consistent with the findings of the present study. However, we should conduct a multivariate analysis in the future to confirm these findings. In the case of subjective physical function, however, the findings regarding the association with SCD are inconsistent. Although there are reports on the association between subjective physical function and SCD [28,29], there are also reports stating that there is no relationship [26]. However, our findings cannot explain the lack of a relationship between SCD and subjective physical function, thus warranting the need for more studies.

According to our findings, it is clear that SCD in Japanese, community-dwelling older adults is affected by depressive symptoms and subjective mental function. Thus, alleviating depressive symptoms and subjective mental function decline may help prevent the development of SCD in older Japanese adults. Therefore, based on previous intervention research reports, the following two methods may be applied to prevent and manage dementia in older Japanese adults with SCD:

(i) Multidomain lifestyle intervention, comprising exercise and dietary guidance, cognitive training, social participation, and individualized health guidance to alleviate depressive symptoms [54].
(ii) Group psychological intervention (goal management, strategies for improving metacognition, relaxation training, and psychagogy [55]) and cognitive training [56], to improve subjective mental function.

However, more studies should be conducted to evaluate the effects of these interventions on older Japanese adults with SCD.

This study has several limitations:

(i) The KCL-CF scores were high only in a small number of subjects. In the future, it will be necessary to have larger sample sizes and thus investigate the effects of subjective and objective mental and physical functions on SCD severity.
(ii) The only tool used for the evaluation of SCD was a self-reporting questionnaire. The biomarker tests needed to define APOEε4 genotypes and preclinical AD were not performed [57]. In the future, it will be necessary to perform biomarker tests to evaluate SCD and thus judge whether similar results will be obtained.
(iii) We used a cross-sectional design; thus, the causality of the relationships found cannot be ascertained. To clarify the relationships between changes in each index and SCD onset, a longitudinal study will be necessary in the future.

Despite these limitations, the results of this study provide valuable information for healthcare professionals in the geriatric field and can be useful for the design of a dementia prevention intervention program for older adults.

## 5. Conclusions

Depressive symptoms and decreased subjective mental function were found to be associated with SCD onset in Japanese, community-dwelling older adults. This suggests that healthcare professionals in the geriatrics field must implement measures to alleviate depressive symptoms through complex interventions combining physical activity, lifestyle improvement, etc., and to improve subjective mental function through psychagogy and cognitive training. Despite the limitations of this study, these findings will be useful for the design and selection of dementia prevention and intervention programs by Japanese healthcare professionals in the geriatrics field.

## Figures and Tables

**Table 1:** KCL-CF scores and frequency data for SCD items.

Variable	Number of Subjects/Frequencies
KCL-CF score (0/1/2/3; *n*)	184/78/22/1
**Frequency of subjective memory item endorsement**	
Do your family or your friends point out your memory loss?	15.1%
Do you make a call by looking up phone numbers?	2.1%
Do you find yourself not knowing today’s date?	26.7%

KCL-CF: Kihon Checklist-Cognitive Function. SCD: subjective cognitive decline. *n*: number.

**Table 2:** Comparison of fundamental information and measurements between the SCD and non-SCD groups.

Variable		Total (*n* = 285)			SCD (*n* = 101)			Non-SCD (*n* = 184)			Effect Size (*r*, *V*)	*p*	
Attribute	Age (yr)	73.3	±	6.3	73.7	±	6.2	73.1	±	6.3	0.05	0.444	
	Sex: Male/female	51/234			16/85			35/149			0.04	0.628	a
	Height (cm)	153.0	±	7.9	152.7	±	7.5	153.2	±	8.1	<0.01	0.981	
	Weight (kg)	52.6	±	9.4	51.6	±	8.7	53.1	±	9.7	0.08	0.201	b
	BMI (kg/m^2^)	22.4	±	3.3	22.1	±	3.1	22.6	±	3.3	0.07	0.222	b
	Educational history (yr)	11.6	±	2.1	11.3	±	1.9	11.7	±	2.2	0.09	0.137	
Objective mental function	MMSE (score)	27.6	±	2.2	27.3	±	2.4	27.8	±	2.1	0.12	0.049	
	GDS-5 (score)	0.6	±	1.0	1	±	1.2	0.4	±	0.8	0.26	<0.001	
Objective physical function	TUG (s)	6.1	±	1.7	6.2	±	1.2	6.1	±	1.9	0.12	0.044	
	Knee extension strength (kg)	17.8	±	5.7	16.6	±	5.2	18.4	±	5.8	0.15	0.012	
Subjective mental function	MCS	52.2	±	4.9	50.7	±	5.9	53	±	4.1	0.18	0.003	
Subjective physical function	PCS	48.7	±	6.2	48.2	±	6.4	49	±	6.1	0.08	0.181	

Data are presented as mean ± standard deviation. SCD group: KCL-CF ≥ 1; non-SCD group: KCL-CF = 0. Mann–Whitney. U-test using independent samples. a: χ^2^ test; b: t-test using independent samples. Yr: year; BMI: body mass index; cm: centimeter; kg: kilogram; MCS: mental component summary; PCS: physical component summary; SCD: subjective cognitive decline; TUG-timed up-and-go test; MMSE: Mini-Mental State Examination; GDS-5: five-item version of the Geriatric Depression Scale.

**Table 3:** Logistic regression analysis with the presence or absence of SCD as the dependent variable.

Variable		B	S.E.	Wald	*p*	Exp (B)	95% CI for Exp (B)	
							Lower	Upper
Attribute	Age (yr)	−0.010	0.028	0.128	0.720	0.990	0.937	1.046
	Sex	0.172	0.382	0.203	0.652	1.188	0.562	2.512
	Educational history (yr)	−0.027	0.077	0.122	0.727	0.974	0.838	1.131
Objective mental function	MMSE	−0.108	0.069	2.432	0.119	0.898	0.784	1.028
	GDS-5	0.474	0.159	8.865	0.003	1.607	1.176	2.196
Objective physical function	TUG	−0.099	0.108	0.841	0.359	0.906	0.734	1.119
	Knee extension strength	−0.026	0.029	0.760	0.383	0.975	0.920	1.033
Subjective mental function	MCS	−0.084	0.031	7.496	0.006	0.919	0.866	0.976
Subjective physical function	PCS	−0.032	0.024	1.736	0.188	0.968	0.923	1.016

B: unstandardized coefficient; S.E.: standard error; Yr: year; CI: confidence interval; MCS: mental component summary; PCS: physical component summary; SCD: subjective cognitive decline; TUG: timed up-and-go test; MMSE: Mini-Mental State Examination; GDS-5: five-item version of the Geriatric Depression Scale.
